# DNA repair goes hip-hop: SMARCA and CHD chromatin remodellers join the break dance

**DOI:** 10.1098/rstb.2016.0285

**Published:** 2017-08-28

**Authors:** Magdalena B. Rother, Haico van Attikum

**Affiliations:** Department of Human Genetics, Leiden University Medical Center, Einthovenweg 20, 2333 ZC Leiden, The Netherlands

**Keywords:** DNA double-strand break (DSB), DSB signalling, DSB repair, chromatin remodellers, SMARCA, CHD

## Abstract

Proper signalling and repair of DNA double-strand breaks (DSB) is critical to prevent genome instability and diseases such as cancer. The packaging of DNA into chromatin, however, has evolved as a mere obstacle to these DSB responses. Posttranslational modifications and ATP-dependent chromatin remodelling help to overcome this barrier by modulating nucleosome structures and allow signalling and repair machineries access to DSBs in chromatin. Here we recap our current knowledge on how ATP-dependent SMARCA- and CHD-type chromatin remodellers alter chromatin structure during the signalling and repair of DSBs and discuss how their dysfunction impacts genome stability and human disease.

This article is part of the themed issue ‘Chromatin modifiers and remodellers in DNA repair and signalling’.

## 1. Introduction

Our cells are exposed to various deleterious agents causing tens of thousands of lesions in the genome every day [1]. Of all the different DNA lesions that occur, DNA double-strand breaks (DSBs) are among the most toxic. If left unrepaired or repaired inaccurately, DSBs can lead to mutations and chromosomal translocations, thereby increasing predisposition to various human disorders such as cancer [2]. DSBs can be produced by exogenous sources such as ionizing radiation (IR) from cosmic radiation and medical treatments, or by chemical compounds such as bleomycin and cisplatin used in cancer chemotherapy. DSBs can also be formed as by-products of intracellular metabolic activities producing reactive oxygen species (ROS), by the stalling and collapse of DNA replication forks and somatic recombination of antigen receptor loci [3–5]. Eukaryotic cells have evolved mechanisms collectively termed the DNA damage response (DDR) that detect, signal and repair DNA lesions such as DSBs to prevent genomic instability and human disease [2].

## 2. Detection and signalling of DNA double-strand breaks

The detection and signalling of DSBs involves the MRE11-RAD50-NBS1 (MRN) and Ku70-Ku80 (Ku) complexes, which bind DSBs and recruit the ATM and DNA-PKcs kinases, respectively [6,7]. ATM, and to a lesser extent DNA-PKcs, subsequently phosphorylates histone H2AX, forming γH2AX, which spreads across up to 2 Mb large chromatin domains surrounding DSBs [8,9]. γH2AX serves as a loading platform for MDC1, which physically associates with the ATM- and DNA-PKcs-induced phospho-mark on H2AX [10]. ATM then phosphorylates MDC1, which allows it to recruit RNF8. This ubiquitin E3 ligase ubiquitylates histone H1, which promotes accrual of the RNF168 ubiquitin E3 ligase. RNF168 further decorates DSB-flanking chromatin with ubiquitin conjugates, thereby promoting the assembly of the BRCA1-Abraxas-RAP80-MERIT40 (BRCA1-A) complex and 53BP1 protein, which regulate DSB repair (see below).

## 3. DNA double-strand break repair by homologous recombination and non-homologous end-joining

Cells use two main pathways for the repair of DSBs: non-homologous end-joining (NHEJ) and homologous recombination (HR) [2,11,12]. During NHEJ, the broken ends are recognized by the DNA end-binding protein complex Ku, which consists of Ku70 and Ku80, and recruits, binds and activates DNA-PKcs. Ku and DNA-PKcs recruit and activate several enzymes involved in the processing of DNA ends such as the Artemis nuclease and DNA polymerases μ and λ. Finally, the broken ends are sealed by a ligase complex composed of XLF-XRCC4-DNA ligase IV. Repair via NHEJ can occur in an error-free or error-prone manner. Unprocessed DNA ends are usually re-ligated without errors, whereas limited end-processing may introduce deletions, and polymerase-dependent filling of gaps may lead to insertions at the repair site [11].

HR on the other hand is initiated by the MRN complex, which recognizes and binds broken DNA ends. MRN acts in concert with CtIP to initiate DNA end-resection, a process that leads to the formation of 3′ single-stranded (ss) DNA overhangs. Further resection of the ends is mediated by the EXO1 and DNA2 nucleases. The stretches of ssDNA are covered and stabilized by RPA, which allows for recruitment of the BRCA1-PALB2-BRCA2 complex. Finally, BRCA2 facilitates removal of RPA and loading of RAD51 recombinase, which searches for an undamaged homologous template, usually a sister chromatid. Following strand invasion and duplication of the sister chromatid, the DSB is repaired in an error-free manner [12].

NHEJ and HR are tightly regulated throughout the cell cycle. While NHEJ is dominant in G1 and late G2 phase [13], HR occurs predominantly in S and early G2 phase [13]. The choice between HR and NHEJ during the cell cycle is regulated through the concerted action of the E3 ubiquitin ligases RNF8 and RNF168, which promote the ubiquitin-dependent recruitment of the BRCA1-Abraxas-RAP80-MERIT40 (BRCA1-A) complex and 53BP1 protein [14–16]. It was recently described that ZMYM3 links BRCA1 to damaged chromatin through specific interactions with RAP80, thereby sequestering BRCA1 away from the HR repair site. This limits its availability for the BRCA1-PALB2-BRCA2-RAD51 complex and consequently impacts BRCA1-driven HR in S and G2 phase [16–18]. In addition, BRCA1's interaction with PALB2 is blocked in G1 due to suppressive ubiquitylation of the BRCA1-interacting domain in PALB2, impairing HR by reducing BRCA1-PALB2-BRCA2-RAD51 complex formation [19]. Finally, it was shown that in G1 phase TIRR-regulated recruitment of 53BP1 to DSBs inhibits DNA end-resection through 53BP1's effectors RIF1 and MAD2L2, thereby impairing HR and favouring NHEJ [14,20–22]. In S and G2 phase, however, RIF1 accumulation at DSB sites is strongly antagonized by BRCA1 and its interacting partner CtIP, allowing efficient end-resection and HR to occur [14,23]. Thus, the interplay between distinct pathways operating at DSBs dictates the mechanism by which these lesions are repaired throughout the cell cycle. For a more detailed overview of the pathways operating at DSBs, we refer to some excellent recent reviews [24–26].

## 4. Chromatin remodelling and the DNA double-strand break response

DNA is packaged through histone and non-histone proteins into a higher order structure called chromatin [27]. Consequently, the response to DSBs has to occur in chromatin, which raises the question as to how DSBs can be detected, signalled and repaired within this context. Chromatin structure is tuned by various processes such as DNA methylation [28], histone post-translational modifications (PTMs) [29,30], as well as nucleosome remodelling by ATP-dependent chromatin remodelling complexes [31,32]. Chromatin remodelling is carried out by large protein complexes that modulate chromatin structure by sliding nucleosomes along DNA, ejecting histone octamers, or ejecting and replacing histone dimers with dimers containing histone variants [31,32]. All chromatin remodelling complexes contain an ATPase/helicase of the SWI2/SNF2 (switch/sucrose non-fermenting) superfamily that generates the energy for chromatin remodelling through the hydrolysis of ATP. The presence of additional distinct functional domains in the ATPase/helicase proteins allows for the classification of chromatin remodelling complexes into four distinct subfamilies: SWI/SNF, ISWI (imitation switch), CHD (chromodomain helicase DNA-binding) and INO80 (inositol 80). SWI/SNF members contain a bromodomain, which binds acetylated histones and a helicase-SANT-associated (HSA) domain for DNA binding. ISWI remodellers contain HAND, SANT and SLIDE domains, which allow for DNA binding within nucleosomes. CHD remodellers also possess a domain for DNA binding, but additionally contain a chromodomain that recognizes methylated histones. Finally, INO80 remodellers do not contain any specific histone-binding motifs and their ATPase region is unique in that it contains an insertion that splits it in two segments [31,32].

It has become clear that both histone PTMs such as phosphorylation (e.g. γH2AX), acetylation and methylation, as well as ATP-dependent chromatin remodelling play important roles in modulating chromatin structure during the DSB response [29,30,32,33]. Here we provide an overview of the current knowledge about the role of ATP-dependent chromatin remodelling complexes in the DSB response. In particular, we will describe the role of SMARCA- and CHD-type remodellers in the DSB response, focusing on the SMARCA2 (BRM), SMARCA4 (BRG1), SMARCA5 (SNF2H), SMARCAD1, CHD1, ALC1 (CHD1-like), CHD2, CHD3 and CHD4 ATPases (table 1).

**Table 1.** Function of chromatin remodellers in the signalling and repair of DSBs.

name	DSB signalling		HR		NHEJ	
	function	reference	function	reference	function	reference
SMARCA2	γH2AX spreading H3 acetylation	[38]	RAD51 loading	[40]	Ku loading	[35,40,71]
SMARCA4	γH2AX spreading H3 acetylation	[38]	RAD51 loading	[37,40]	Ku loading	[35,40,71]
SMARCA5	RNF168 response BRCA1 loading	[41]	RPA and RAD51 loading	[74,75]	Ku loading	[73]
					CENP-S and CENP-X deposition (?)	[46,76]
					repair of heterochromatic DSBs	[77]
INO80			histone H2A.Z removal, Mre11/Sae2-dependent end-resection	[51,55,83,94]	histone H3 removal	[90]
Fun30/SMARCAD1			passing the Rad9 (53BP1) chromatin barrier for EXO1/DNA2-dependent end-resection	[47,49,80]		
CHD1			CtIP loading	[57]		
ALC1					association with Ku70 and DNA-PKcs	[83]
CHD2					chromatin expansion, H3.3 deposition XRCC4 and DNA ligase IV loading	[58]
CHD3					repair of heterochromatic DSB repair	[92]
CHD4	RNF8 response BRCA1 loading	[93–95]	RPA and RAD51 loading	[62,93–94,101]		

## 5. The SMARCA class of chromatin remodellers

Several SWI/SNF2 family members are known by a SMARCA (SWI/SNF-related, Matrix-associated, Actin-dependent Regulator Chromatin group A) acronym, classifying them in the SMARCA class of chromatin remodellers. This concerns SMARCA1 (SNF2L), SMARCA2 (BRM), SMARCA3 (LTF), SMARCA4 (BRG1), SMARCA5 (SNF2H), SMARCA6 (HELLS), SMARCAD1 and SMARCAL1, which belong to three different SWI/SNF2 subfamilies: SWI/SNF, ISWI and INO80. SMARCA chromatin remodellers act in different multi-protein complexes that play important roles in several distinct biological processes such as transcription, cell division and development, as well as DNA repair [31,32]. Here we highlight the function of SMARCA2, SMARCA4, SMARCA5 and SMARCAD1 in the DSB response (table 1).

## 6. SMARCA2 and SMARCA4

SMARCA2 (BRM) and SMARCA4 (BRG1) were originally identified as Snf2 and Sth1, respectively, in *Saccharomyces cerevisiae* [31]. Biochemical and cellular studies in yeast revealed that Snf2 and Sth1 are the catalytic subunits of the multi-protein SWI/SNF and ‘remodel the structure of chromatin’ (RSC) complexes, respectively, that possess ATP-dependent chromatin remodelling activity. Mutations in subunits of the SWI/SNF (e.g. Snf2 and Snf5) and RSC (e.g. Rsc1, Rsc2, Sfh1 and Sth1) chromatin remodelling complexes rendered cells hypersensitive to DSB-inducing agents [63–65], suggesting a role in the DSB response. Indeed, several subunits of SWI/SNF (e.g. Swi2, Snf5) and RSC (e.g. Sth1 and Rsc8) were shown to be recruited to a nuclease-induced DSB [63,65,66]. Interestingly, different mechanisms appeared to regulate the recruitment of these chromatin remodelling complexes to DSBs during HR and NHEJ. Specifically, the NuA4 and Gcn5 enzymes were found to be required for efficient recruitment of SWI/SNF to DSBs, where it promotes phosphorylation of H2AX and the strand invasion step of HR (particularly in the context of heterochromatin) [65–68]. RSC recruitment on the other hand was dependent on the Mre11 (involved in both HR and NHEJ) and Ku70 proteins (unique to NHEJ) and probably involves its physical association with these proteins [63,65]. Consistently, RSC facilitates HR by directing nucleosome sliding during end-resection and by promoting extension of the invading strand [63,65,69]. RSC was also found to promote homologous recombination between sister chromatids by promoting cohesin loading at DNA breaks [70]. Moreover, while one study showed that RSC mutants were competent for NHEJ [65], another study showed that loss of functional RSC renders cells NHEJ-deficient [63]. Although this latter study implicates a role for RSC in NHEJ, it is still unclear how its chromatin remodelling activity contributes to this repair process.

**Figure 1.**
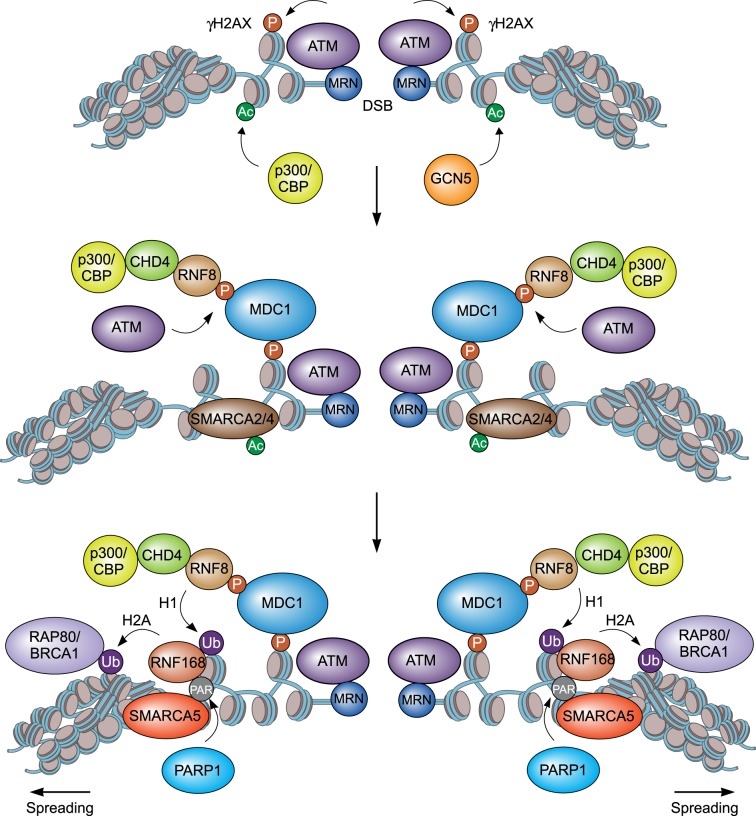
Model for the role of chromatin remodellers in DSB signalling. SMARCA2 and SMARCA4 are recruited to the DSB and modify chromatin to enable spreading of ATM-induced γH2AX and enhance the acetylation response around the lesion. Subsequently, SMARCA5 and CHD4 remodellers arrive at the break and promote RNF8/RNF168-dependent ubiquitin signalling, leading to the assembly of RAP80-BRCA1. This involves interactions between RNF8 and CHD4, and RNF168 and SMARCA5, which are stimulated by p300/CBP and PARP1, respectively.

The human counterparts of the yeast SWI/SNF and RSC complexes are called BAF (BRG1-Associated Factors) and PBAF (Polybromo-Associated BAF) [31]. SMARCA2 and SMARCA4 are mutually exclusive ATPases in the BAF complex, whereas only SMARCA4 is found in the PBAF complex. SMARCA2 and SMARCA4 are both recruited to DSBs in a manner dependent on the phosphorylation and acetylation of histones [9,34,36,38]. DSB-activated ATM establishes low levels of γH2AX in DSB-flanking chromatin. This leads to the recruitment of histone acetyltransferases such as GCN5, which triggers the acetylation of histone H3 within these nucleosomes. SMARCA4 can bind these nucleosomes by interacting with acetylated H3 via its bromodomain, and this interaction is enhanced by its ATM-dependent phosphorylation on Ser721 [36,68]. SMARCA2 recruitment to DSB does not rely on GCN5, but instead requires CBP/p300-dependent acetylation of DSB-flanking chromatin [68,71]. SMARCA2 and SMARCA4 increase chromatin accessibility around the DSBs by sliding nucleosomes and ejecting histones [31], thereby allowing spreading of γH2AX throughout the damaged chromatin compartment and acetylation of histone H3 at the break to promote an efficient DSB response [38,68] (figure 1). Thus, H2AX phosphorylation and histone acetylation mediate DSB recruitment of SMARCA2 and SMARCA4, which remodel DSB-flanking chromatin for the proper assembly of DSB repair proteins.

**Figure 2.**
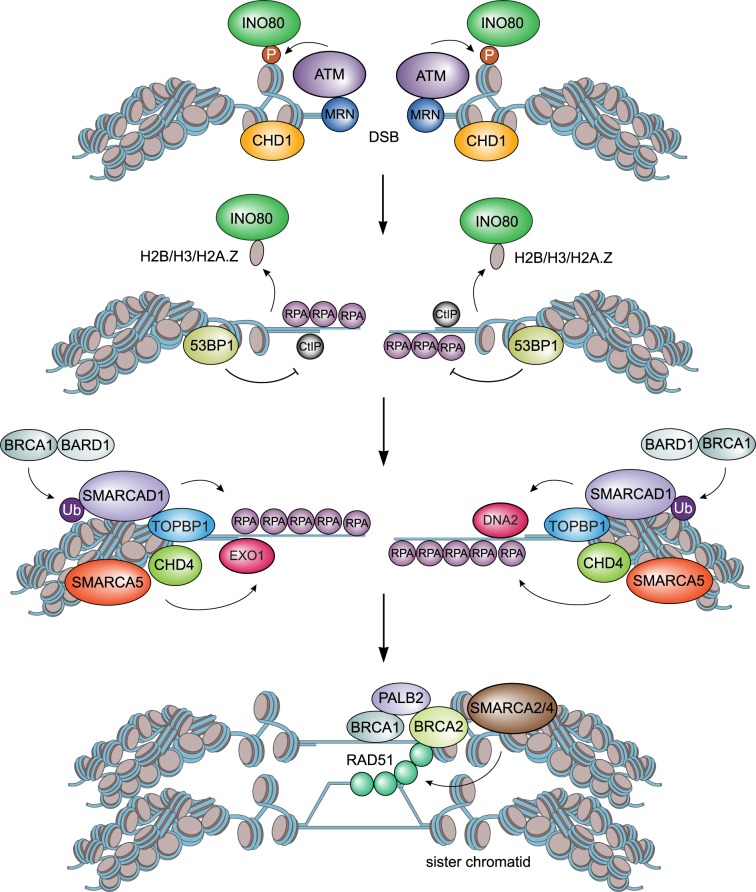
Model for the role of chromatin remodellers in HR-mediated DSB repair. INO80 is recruited to the DSB and ejects histones H2B/H3/H2A.Z in close proximity to the lesion. Subsequently, CHD1 is recruited and opens chromatin around the DSB to promote CtIP loading and initiate DNA end-resection. Next, SMARCAD1 recruitment aids in passing the 53BP1-induced chromatin barrier and stimulating EXO1 and DNA2-dependent end-resection for efficient assembly of HR factors (BRCA1, PALB2, BRCA2 and RAD51), the latter of which is facilitated by SMARCA2, SMARCA4, SMARCA5 and CHD4 chromatin remodelling.

**Figure 3.**
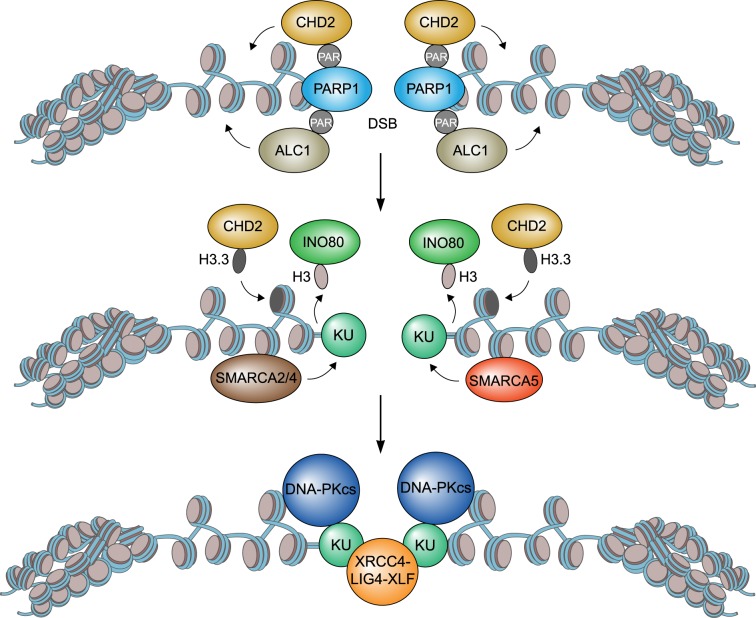
Model for the role of chromatin remodellers in NHEJ-mediated DSB repair. CHD2 and ALC1 are recruited to the DSB by PARP1 and expand chromatin around this lesion, whereas INO80 recruitment leads to histone eviction. CHD2 also deposits variant histone H3.3. Ku recruitment is favoured by CHD2, SMARCA2, SMARCA4 and SMARCA5 chromatin remodelling, leading to XRCC4-LIG4 assembly and efficient NHEJ.

SMARCA2 and SMARCA4 have been implicated in DSB repair. However, the exact mechanism by which these chromatin remodellers affect this process is not completely understood due to conflicting findings. One study demonstrated that SMARCA4 facilitates HR rather than NHEJ as it was shown to specifically promote RAD51-mediated DNA strand invasion by regulating the replacement of RPA with RAD51 on ssDNA [37]. Two other reports, however, showed that the SMARCA2- and SMARCA4-containing BAF and PBAF complexes are particularly involved in NHEJ [35,39,72]. While BAF was shown to promote NHEJ by facilitating the accrual of Ku at DSBs [35,39,71]. PBAF on the other hand was shown to repress transcription in the vicinity of DSBs [72], a process that was proposed to be required for Ku loading at these lesions [42]. However, precisely how PBAF affects NHEJ by promoting transcriptional repression and subsequent Ku assembly remains to be established. Finally, yet another report implicated SMARCA4, as well as SMARCA2, in both NHEJ and HR [40]. SMARCA2 and SMARCA4, together with the BAF170, BAF155 and SF5 proteins, are part of a complex that is recruited to DSBs in manner dependent on BRIT1. Loss of BRIT1 causes impaired chromatin relaxation at DSBs due to a decrease in the association of SMARCA2 and SMARCA4 with these lesions. This negatively impacts the loading of DSB repair factors such as RAD51 and Ku70, and consequently impairs both HR and NHEJ [39,40,68] (figures 2 and 3). Thus, while it is evident that SMARCA2 and SMARCA4 impact DSB repair, future studies are required to clarify the exact mode of action of these chromatin remodellers during HR and NHEJ.

## 7. SMARCA5

SMARCA5 (SNF2H) is a member of the ISWI family of chromatin remodellers, which was first purified from *Drosophila melanogaster*. It is the catalytic subunit of the CHRAC, ACF, RSF, WHICH and NoRC chromatin remodelling complexes in mammalian cells. SMARCA5 has the ability to remodel chromatin by sliding and displacing nucleosomes [31,32]. It also rapidly recruits to DNA damage sites generated by laser micro-irradiation, as well as to bona fide, nuclease-induced DSBs [41,73,74]. Different mechanisms have been described to contribute to the efficient accumulation of this chromatin remodeller at DNA breaks. First, SMARCA5 interacts with ACF1 (another subunit of the CHRAC and ACF complexes) through its SLIDE domain and this interaction drives its accrual to DSBs [41,73]. Second, poly(ADP-ribosyl)ation by poly(ADP-ribose) polymerase 1 (PARP1) was shown to trigger both the accumulation and spreading of SMARCA5 at these DNA lesions [41] (figure 1). Third, SMARCA5 recruitment to DSBs requires the RNF20-RNF40 E3 ubiquitin ligase complex. RNF20-RNF40 mono-ubiquitylates histone H2B, which in turn leads to the methylation of Lys4 in histone H3, a PTM that is recognized by SMARCA5 [43,44,75]. However, it should be noted that another study did not observe increased methylation of Lys4 in histone H3 as a consequences of DNA damage-induced RNF20-RNF40-dependent mono-ubiquitylation of histone H2B, questioning the relevance if this PTM for SMARCA5 recruitment to DSBs [45]. Fourth and last, SIRT6, which deacetylates histone H3K56, was shown to recruit SMARCA5 to DSBs, although it is unclear how loss of this specific PTM affects this event [74]. Equally unclear is whether crosstalk between these four distinct mechanisms exists and contributes to the efficient accrual and functioning of SMARCA5 at DNA breaks.

SMARCA5 has been implicated in the RNF168 signalling cascade at DSBs. It interacts physically with RNF168 in the DSB-flanking chromatin compartment in a PARP1-dependent manner. In fact, PARP1 PARylates RNF168 and PARylated RNF168 is bound by SMARCA5. RNF168-bound SMARCA5 is then thought to locally remodel chromatin, thereby facilitating further RNF168-dependent histone ubiquitylation and ultimately BRCA1 accumulation at DSBs (figure 1). These findings suggest a feed-forward loop process in which PARP1 couples SMARCA5 chromatin remodelling to RNF168 to drive the ubiquitylation of damaged chromatin and the subsequent assembly of BRCA1 [41,74,75].

SMARCA5 affects DSB repair by HR and NHEJ and protects cells against DSB-inducing agents [41,73–75]. Loss of SMARCA5 has been shown to impair the accumulation of HR factors such as RPA and RAD51 at DSBs, suggesting that it affects HR by regulating end-resection [74,75] (figure 2). However, how SMARCA5 chromatin remodelling activity promotes end-resection remains to be established. The role of SMARCA5 in NHEJ, on the other hand, has been studied in more detail and was shown to depend on ACF1 [73]. ACF1 recruits components of the ACF and CHRAC complexes including SMARCA5. ACF1 also interacts with Ku70-Ku80 to facilitate Ku binding to DSBs [39,68,73]. This latter event, however, was also dependent on SMARCA5's chromatin remodelling activity, suggesting that chromatin structural changes induced by SMARCA5 promote efficient ACF1-dependent loading of Ku at DSBs, thereby facilitating repair of these lesions via NHEJ (figure 3).

Another SMARCA5-interacting protein that may support its function in DSB repair is Remodelling and Spacing Factor 1 (RSF1), with which it forms the RSF complex. Indeed, one report showed that SMARCA5 and RSF1 cooperate to promote NHEJ by depositing the centromeric histone-fold proteins CENP-S and CENP-X at DSBs. These CENP proteins in turn recruit and regulate the mono-ubiquitination of the Fanconi Anaemia proteins FANCD2 and FANCI [76]. Mono-ubiquitinated FANCD2 and FANCI presumably function as docking site for the accrual of DSB repair proteins, although this remains to be studied in more detail. Another study, however, showed that RSF1 facilitates the loading of CENP-S and CENP-X in a manner independent of SMARCA5. This work further showed that the recruitment of CENP-S and CENP-X facilitates the assembly of NHEJ factors such as XRCC4, providing a likely explanation for how RSF1 regulates NHEJ [46]. Precisely how the CENP-S and CENP-X proteins regulate NHEJ and whether this involves chromatin structural changes or direct interactions with the NHEJ machinery remains to be established. Finally, SMARCA5 was also shown to promote the Artemis-dependent repair of DSBs in heterochromatin. Here SMARCA5, in concert with ACF1, promotes chromatin relaxation following dispersal of the repressive CHD3 chromatin remodeller [9,39,77]. This way SMARCA5-ACF1 provide access to the damaged DNA for Artemis, allowing this nuclease to process the broken DNA and stimulate NHEJ-dependent repair. A more detailed description of CHD3's function in DSB repair is provided in another section of this review (see below).

## 8. Fun30/SMARCAD1

Human SMARCAD1 and its orthologue in yeast, Fun30, belong to the INO80 family of chromatin remodellers, whose founding member Ino80 was first discovered in *Saccharomyces cerevisiae* [31]. INO80 was shown to play important roles in transcription regulation, mitosis and DSB repair [51–54,78,79]. More recently, it became evident that SMARCAD1 and Fun30 are also critical for transcription regulation and DSB repair [47–49,80,81]. Fun30, similar to INO80, is rapidly recruited to DSBs in a manner dependent on the Dbp11 scaffold protein and the 9-1-1 complex, which senses DNA damage and is composed of the Ddc1, Mec3 and Rad17 proteins [47,48,80,82]. At DSBs it changes chromatin structure to promote resection of the broken DNA. However, while INO80 chromatin remodelling initiates Mre11/Sae2-dependent end-resection by removing histones such as H2B, H3 and H2A.Z [51,54,55,79,83,84], Fun30 was shown to regulate Exo1- and Dna2-dependent long-range end-resection by relieving the repressive impact of the chromatin-bound Rad9 checkpoint protein on this process [47,48,80] (figure 2). The activity of Fun30 during end-resection in S/G2 phase is controlled in a cell cycle-specific manner through interactions with Dbp11 and the 9-1-1 complex. Another layer of control to this process is provided by cyclin-dependent kinase Cdk1 and the cyclins Clb2 and Clb5, which are recruited to DSBs where they stimulate Fun30 activity by its phosphorylation at Ser28 [56]. Finally, Fun30's role in regulating end-resection was shown to be critical for efficient checkpoint signalling and HR-dependent repair of DSBs [47,48,80].

SMARCAD1, similar to Fun30, is also rapidly recruited to laser micro-irradiation and nuclease-induced DSBs to promote DNA end-resection and DSB repair by HR [47,49]. DSB recruitment of SMARCAD1 is facilitated by the TOPBP1 scaffold protein (orthologue of yeast Dbp11) and BRCA1-BARD1 ubiquitin ligase complex, which decorates histone H2A with ubiquitin [49,56] (figure 2). Mechanistically, it was shown that SMARCAD1 binds H2A-ubiquitin through its two N-terminal ubiquitin-binding CUE domains [49]. Subsequently, SMARCAD1's chromatin remodelling activity facilitates the repositioning of 53BP1 (orthologue of yeast Rad9) at the DSB [49]. This way SMARCAD1 aids in passing the 53BP1-induced chromatin barrier and stimulating end-resection for efficient assembly of RPA and RAD51 during HR [47,49] (figure 2).

## 9. The CHD class of chromatin remodellers

The CHD (chromodomain helicase DNA-binding) family of chromatin remodellers was originally identified in *Xenopus laevis* [31]. The family consists of nine members subdivided into three classes: class I (CHD1 and CHD2), class II (CHD3, CHD4 and CHD5) and class III (CHD6, CHD7, CHD8 and CHD9) [85,86]. A general feature of the family is that all catalytic subunits contain two N-terminal, tandemly arranged chromodomains, which bind to methylated histones and DNA, and an ATPase/helicase domain of the SWI2/SNF2 superfamily located in their central region [31]. The class I CHD proteins additionally contain a C-terminal DNA binding domain that has affinity for AT-rich sequences, allowing them to directly bind to DNA. The class II CHD enzymes also possess N-terminal paired PHD Zn-finger-like domains involved in the recognition of methylated histone tails. Finally, class III CHD proteins additionally contain a C-terminal BRK (Brahma and Kismet) region whose function is still ambiguous [86]. Up to now, a role in the DSB response has been established for CHD1, a CHD1-related chromatin remodeller known as CHD1-like or ALC1, CHD2, CHD3 and CHD4, which will be discussed below (table 1).

## 10. CHD1

A recent study showed that CHD1 is recruited to chromatin in response to DSB induction and that it is required for HR-mediated repair of DSBs [57]. CHD1 recruitment was dependent on MRE11 activity, but not on CtIP or PARP1. Thus, CHD1 was thought to act downstream of the MRN complex and upstream of CtIP in this repair process. Indeed, CHD1 promoted the loading of CtIP onto damaged DNA (figure 2). Accordingly, loss of CHD1 resulted in defects in CtIP-dependent DNA end-resection as manifested by a decrease in the assembly of RPA and RAD51 at DNA breaks. Importantly, formaldehyde-assisted isolation of regulatory elements (FAIRE), which can assess open chromatin status or nucleosome depletion, revealed that CHD1's ATPase activity plays a role in chromatin opening at DSBs. This suggests that CHD1 chromatin remodelling drives HR by facilitating end-resection. However, precisely how CHD1's activity remodels chromatin at DSB remains to be resolved. Interestingly, while CHD1 promotes the HR-mediated repair of DSBs, it did not affect DSB repair by NHEJ as measured using an end-joining-specific reporter assay [57], suggesting that its remodelling activity is specifically required at the early stages of HR.

## 11. CHD1-like (ALC1)

ALC1 (amplified in liver cancer 1) is an ATP-dependent chromatin remodeller that is often classified as a CHD family member despite the absence of an identifiable chromodomain. Instead biochemical and structural analyses revealed a distinct C-terminal macrodomain [87,88]. This domain was shown to mediate the PARP1-dependent recruitment of ALC1 to DNA breaks by binding to DNA damage-associated PAR moieties [50,88,89] (figure 3). Moreover, protein-protein interaction studies revealed that ALC1 associates with DNA repair factors such as Ku70 and DNA-PKcs in a PARP1-dependent manner [88]. Interestingly, recent findings showed that ALC1's ATPase activity is required for the PARP1-dependent expansion of chromatin at DNA damage sites [50]. Collectively, these findings support a role for the PARP1-ALC1 axis in driving chromatin remodelling during DNA repair, probably NHEJ (figure 3). Cells depleted for ALC1 indeed showed increased sensitivity to DSB-inducing agents such as IR [50,88], yet whether and how ALC1's remodelling activity promotes NHEJ and/or other PARP1-dependent repair mechanisms remain to be elucidated.

## 12. CHD2

A recent study demonstrated an important role for the CHD2 chromatin remodeller in the DSB response [58]. CHD2 rapidly accumulates at DSB sites in a manner dependent on PARP1 activity (figure 3). Interestingly, a novel C-terminal domain in CHD2 was identified that binds PAR moieties associated with PARP1, and thereby mediates the PARP1-dependent recruitment of CHD2 to DSBs. CHD2 chromatin remodelling activity, similar to the activity of ALC1, was found to promote local chromatin expansion at DNA damage sites. However, whether ALC1 and CHD2 act synergistically during this process remains to be resolved. In addition, it was shown that CHD2, which deposits histone variant H3.3 at sites of active transcription and at sites of UV-C-induced DNA damage [59,60], also facilitates the incorporation of this histone variant in DSB-flanking chromatin, potentially in concert with the histone chaperones CAF-1 and HIRA [58,61,90]. Given that H3.3 incorporation at DSBs was also dependent on PARP1, the data support a model in which PARP1 recruits the CHD2 chromatin remodeller to DSBs to promote local chromatin expansion and H3.3 incorporation. Mechanistically, it was shown that PARP1, CHD2 and H3.3 regulate the assembly of core NHEJ proteins such as XRCC4 and DNA ligase IV to promote efficient NHEJ [58] (figure 3). Thus, CHD2, as an effector of PARP1, promotes DSB-induced chromatin expansion, deposits histone variant H3.3, and facilitates the efficient recruitment and proper functioning of the NHEJ repair machinery. Remarkably, CHD2 loss did not affect HR [58], suggesting that the chromatin changes it induces at DSBs play an exclusive role during NHEJ. Interestingly, while CHD2 promotes chromatin relaxation and H3.3 incorporation, INO80 was shown to remove histones from these lesions to facilitate NHEJ [90] (figure 3). Future work should resolve how these different chromatin remodelling activities crosstalk during NHEJ.

## 13. CHD3

CHD3 and CHD4 are the mutually exclusive catalytic ATPase subunits of the Nucleosome Remodelling and Deacetylase (NuRD) complex. Additionally, this complex contains the histone deacetylases HDAC1 and HDAC2 as subunits, which help to repress transcription of NuRD target genes by histone deacetylation [61,91]. While CHD3 is also involved in this process, it has also been shown to act during the repair of DSBs in heterochromatin. Repair of DNA breaks in heterochromatin is hampered by the presence of KAP-1, which keeps chromatin compacted. This involves the SUMOylation of KAP-1, a PTM that is recognized and bound by CHD3. However, the formation of DNA breaks in heterochromatin triggers the ATM-dependent phosphorylation of KAP-1 at the Ser824 and the subsequent release of CHD3 [9,61,92]. Biochemically, it was shown that KAP-1 phosphorylation generates a motif that perturbs the interaction of the SUMO-interacting motif in CHD3 with SUMO-KAP-1 [92]. The release of CHD3 enables chromatin relaxation, yet this event alone was insufficient for efficient DSB repair. Additionally, chromatin relaxation driven by the ACF1-SMARCA5 chromatin remodelling complex, which is recruited to heterochromatic DSBs by the RNF20-RNF40 ubiquitin ligase, was required [39,77]. These findings show that a two-step release and recruitment system modulates opposing chromatin remodelling activities during DSB repair in heterochromatic regions.

## 14. CHD4

CHD4 is rapidly recruited to sites of laser- and IR-induced DSBs [62,93–96] and its recruitment to these lesions occurs in a PARP1-dependent manner [95,96] (figure 1). Mechanistically, it was shown that CHD4 can bind PAR chains *in vitro* through an N-terminal domain located between residues 145–225, which is structurally similar to the high mobility group box (HMG) domain [95,97]. Loss of this specific domain impaired CHD4 recruitment, suggesting this domain mediates CHD4 recruitment by binding PARylated PARP at DSB sites. More recently, it was shown that the PARP1-dependent recruitment of CHD4 to damaged chromatin involves ZMYND8. Specifically, the MYND domain in this protein was found to mediate the PARP-dependent recruitment of the GATAD2A/NuRD complex to DNA damage sites [98]. Whether this involves a direct association of ZMYND8's MYND domain with PAR moieties is unclear. Alternatively, given that the MYND domain in ZMYND8 was also shown to be required for its association with the NuRD complex, we cannot rule out the possibility that a subunit other than ZMYND8 interacts with PAR and mediates the PARP-dependent recruitment of NuRD to DSB sites. On the other hand, another report demonstrated that the bromodomain in ZMYND8 drives NuRD recruitment by recognizing TIP60-acetylated histone H4 within damaged chromatin [61,68,99]. However, different ZMYND8 isoforms were studied in these reports [98,99], raising the question as to how these isoforms can be recruited by PAR- and H4 acetylation-dependent mechanisms. Furthermore, CHD4 assembly at sites of DNA damage was dependent on RNF8 and involves a non-canonical interaction between CHD4 and the forkhead-associated domain, but not the catalytic RING domain of RNF8 [93] (figure 1). CHD4 was also shown to interact with the histone acetyltransferases p300 in a manner dependent on PARP1. Interestingly, p300 was found to be required for DSB recruitment of CHD4, as well as its interaction with RNF8 [100] (figure 1). These findings suggest that p300 may play a critical role in the recruitment of CHD4 upstream of RNF8, whereas PARP1 may be important for the interaction between p300 and CHD4 [61,68,93,95,96,100]. Undoubtedly, additional work is needed to unravel how the crosstalk between PARP, p300 and RNF8 affects the accumulation and functionality of the CHD4-containing NuRD complex at DSBs.

CHD4 has been shown to affect both the signalling and repair of DSBs [62,93,94]. The signalling of DSBs involves the RNF8-dependent recruitment of CHD4, which promotes local chromatin decondensation to facilitate RNF8/RNF168-dependent chromatin ubiquitylation and subsequent BRCA1 recruitment [62,93,94] (figure 1). The repair of DSBs by HR is largely dependent on CHD4 and its interaction with BRIT1 [101]. CHD4's chromatin remodelling activity was shown to be required for the recruitment of BRIT1, a key regulator of HR. In fact, loss of CHD4, similar to that of BRIT1, impaired the assembly of RPA, RAD51 and BRCA2 at DSBs and rendered cells hypersensitive to PARP inhibitor-induced DSBs, a key feature of HR-deficient cells [62,93,94,101] (figure 2). The proper loading of HR proteins by CHD4 requires actively transcribed chromatin [102]. Paradoxically, however, the PARP-ZMYND8-CHD4 axis was shown to induce transcriptional silencing in cis at DSBs [61,68,96,99]. This probably involves CHD4's role as a repressive chromatin remodeller [99]. Indeed, recent work implicated an important role for repressive chromatin in the assembly of BRCA1 at DSBs and their subsequent repair by HR [103]. However, whether CHD4 regulates HR by silencing transcription and/or compacting chromatin at DSBs is not fully understood yet and requires further investigation. Finally, CHD4 supports the recruitment of HDAC1 and HDAC2 to sites of DSBs [95]. At DSBs, HDAC1 and HDAC2 facilitate hypoacetylation of H3K56, thereby regulating the persistence of NHEJ factors such as Ku70 and Artemis at the damaged chromatin, and repair of the broken DNA by NHEJ [104]. However, loss of CHD4 itself did not affect the efficiency of NHEJ, raising the question as to how relevant the involvement of CHD4 in recruiting HDAC1 and HDAC2 to DSBs is for DNA repair [100].

## 15. Conclusion and future perspectives

It has become clear that chromatin reorganization during the DSB response is not simply a matter of switching chromatin from a ‘closed’ to ‘open’ state and vice versa. The DSB response is a complex, multi-step procedure, that requires distinct chromatin changes at each step. Only this way cells can properly detect, signal and repair DSBs in the context of chromatin. Evidently, ATP-dependent chromatin remodellers induce chromatin structural changes that are crucial for these DSB responses (table 1).

For instance, SMARCA2 and SMARCA4 act at DSBs to facilitate the ATM-dependent spreading of γH2AX. In addition, CHD4 and SMARCA5 team-up with RNF8 and RNF168, which are recruited in an ATM/γH2AX-dependent manner, to locally unfold chromatin, thereby facilitating ubiquitylation of the damaged chromatin and the recruitment of DNA repair proteins such as BRCA1 to the DSB (figure 1). Thus, it is evident that following the detection of a DSB, several chromatin remodellers engage in the signalling of this lesion by facilitating the assembly of distinct DSB repair proteins. However, it remains to be addressed to what extent these chromatin remodellers contribute to the actual repair of DSBs by HR and/or NHEJ by facilitating this process.

HR requires extensive chromatin remodelling to enable DNA end-resection. To overcome the nucleosomal barrier, INO80 ejects core histones as well as variant histone H2A.Z, while CHD1 ‘opens’ chromatin around DSBs. This facilitates the loading of factors such as MRE11 and CtIP, thereby triggering the initiation of end-resection [57]. Further resection (long-range resection) requires the activity of Fun30/SMARCAD1, which are thought to counteract the repressive impact on chromatin imposed by Rad9/53BP1. This allows the EXO1 and DNA2 nucleases to generate long stretches of DNA within a nucleosomal context, which are subsequently coated by RPA [47,80]. SMARCA5 and CHD4 assist in the loading of RPA, whereas SMARCA2 and SMARCA4 facilitate the replacement of RPA with RAD51 to initiate DNA strand invasion, which is essential for downstream steps of HR [37,40,62,74,75,93,94,101] (figure 2). Thus, several chromatin remodellers cooperate to drive the distinct steps required to execute HR within chromatin. However, our understanding of how chromatin remodellers facilitate the early steps of HR at the molecular level is still quite limited and improving this may require biochemistry, biophysics and structural biology approaches. Moreover, it is also not well understood whether and how at the later stages during HR the homologous chromatin template, which is subject to strand invasion and duplication of the undamaged DNA, is structurally altered by chromatin remodellers.

It has been long thought that NHEJ simply relies on direct sealing of the broken ends without the need of extensive chromatin structural changes. However, recent studies have implicated several chromatin remodellers in NHEJ. For instance, CHD2 and ALC1 work in a PARP1-dependent pathway that facilitates chromatin relaxation [50,58]. Additionally, CHD2 facilitates the incorporation of histone variant H3.3 into damage chromatin. These chromatin structural changes seem to allow for the proper loading of NHEJ factors such as Ku70 and XRCC4, thereby promoting efficient joining of the broken ends [58] (figure 3). However, Ku recruitment is also favoured by SMARCA2, SMARCA4 and SMARCA5, although it is unclear how the activity of these chromatin remodellers contributes to the end-joining process. Future studies may unravel precisely how the activity of chromatin remodellers, as well as their crosstalk at DSBs contribute to NHEJ. Finally, following DSB repair, chromatin has to be restored to its original state in order to preserve a cell's epigenetic status. Given that so many chromatin remodellers act on damaged chromatin, it will be of great interest to unravel how cells restore the original epigenetic code following completion of DSB repair.

Emerging evidence shows that ATP-dependent chromatin remodellers are not only critical for a proper DSB response, but also key in preventing cancer and disorders associated with intellectual disability or ageing. For instance, the inactivation of SMARCA2 and SMARCA4 due to somatic mutations or epigenetic silencing led to the development of various tumours [105]. In contrast, germline mutations in these ATPases have been causally linked to rare intellectual disabilities such as Nicolaides–Baraitser syndrome and Coffin–Siris syndrome [106]. Moreover, somatic mutations in the *CHD2* gene have been associated with lymphoma [107], whereas reduced expression of several NuRD components was observed in the premature aging disease Hutchinson-Gilford progeria syndrome [108]. It is still not completely understood how mutations and expression changes in these chromatin remodellers contribute to disease manifestation. It will therefore be of clinical relevance to further study how their functional contribution to the DSB response relates to disease aetiology. Such knowledge may not only lead to a better understanding of disease mechanisms, but may also pave the way for the development of novel therapeutic regimes that target chromatin remodellers or the defects caused by their loss.

## Data accessibility

This article has no additional data.
